# A new player in circadian networks: Role of electrical synapses in regulating functions of the circadian clock

**DOI:** 10.3389/fphys.2022.968574

**Published:** 2022-11-03

**Authors:** Aishwarya Ramakrishnan Iyer, Vasu Sheeba

**Affiliations:** ^1^ Chronobiology and Behavioural Neurogenetics Laboratory, Neuroscience Unit, Jawaharlal Nehru Centre for Advanced Scientific Research, Bangalore, Karnataka, India; ^2^ Department of Neuroscience and Behavior, Barnard College of Columbia University, New York, NY, United States

**Keywords:** gap junction, circadian, innexin, connexin, neuronal circuit, electrical synapse

## Abstract

Several studies have indicated that coherent circadian rhythms in behaviour can be manifested only when the underlying circadian oscillators function as a well-coupled network. The current literature suggests that circadian pacemaker neuronal networks rely heavily on communication mediated by chemical synapses comprising neuropeptides and neurotransmitters to regulate several behaviours and physiological processes. It has become increasingly clear that chemical synapses closely interact with electrical synapses and function together in the neuronal networks of most organisms. However, there are only a few studies which have examined the role of electrical synapses in circadian networks and here, we review our current understanding of gap junction proteins in circadian networks of various model systems. We describe the general mechanisms by which electrical synapses function in neural networks, their interactions with chemical neuromodulators and their contributions to the regulation of circadian rhythms. We also discuss the various methods available to characterize functional electrical synapses in these networks and the potential directions that remain to be explored to understand the roles of this relatively understudied mechanism of communication in modulating circadian behaviour.

## Introduction

The 24-hour rotation of the earth on its axis has resulted in daily cyclic variations in different biotic and abiotic factors in the environment such as light, temperature, and humidity. To adapt to such daily variations, it is hypothesized that organisms ranging from bacteria to humans evolved to have endogenous timekeeping mechanisms of about 24 h to restrict most of their behavioural and physiological activities to an appropriate time of the day (Nikhil and Sharma, 2017). The central clock in most complex metazoans is located in the brain and is a multi-oscillatory system made up of several different interacting circadian oscillators. Each of these individual oscillators are characterised by a ‘molecular clock’ made up of self-sustaining rhythms of mRNA and proteins that interact to form a Transcriptional-Translational Feedback Loop (TTFL). Although each individual oscillator is a ticking clock by itself, robust circadian rhythms in behaviour at the organismal level are only generated when these individual oscillators function as a network (Beckwith and Ceriani, 2015; Koronowski and Sassone-Corsi, 2021; Micklem and Locke, 2021). A central question in circadian biology therefore deals with understanding how these different oscillators communicate amongst each other to generate coherent rhythms at the molecular, network, and behavioural levels. Multiple approaches—ranging from mathematical modelling to genetic and physiological methods, have been used to understand the functioning of clock networks. At the anatomical and physiological levels, extensive studies have been carried out on different organisms to investigate the various means by which clock cells communicate with each other. Although nervous systems are comprised of both chemical and electrical synapses, most studies on the neuronal regulation of behaviour focus almost exclusively on chemical synapses. In this review, we focus on the latter mode of communication namely, the one mediated by electrical synapses, with a special emphasis on their role in regulating circadian rhythm properties.

## Connectivity in multi-oscillatory circadian networks

Circadian networks in most organisms are made up of multiple oscillators that extensively interact with each other to regulate rhythm properties. As we currently understand them, most of these interactions are mediated by chemical neuromodulators such as neuropeptides and neurotransmitters. Before discussing electrical synapses we will summarize findings from two well-studied multi-oscillatory neuronal networks, the *Drosophila melanogaster* circadian pacemaker network and the mammalian central pacemakers in the Suprachiasmatic nucleus (SCN), to emphasize the importance of connectivity among the oscillators in the network. The central clock in *Drosophila* is made up of ∼150 individual oscillators/neurons distributed bilaterally in the brain, but well-connected with each other (Sheeba, 2008; Ahmad et al., 2021). In *Drosophila,* blocking the synaptic communication in the network by expressing the tetanus toxin light chain (TeTxLC) in all the clock neurons results in about 80%–90% of flies becoming arrhythmic under constant conditions (DD 25°C) and about 60% of flies failing to entrain to Light: Dark cycles (Kaneko et al., 2000), thus underscoring the importance of synaptic transmission. Under constant darkness (DD), the small ventral lateral neurons (s-LNv) are important for maintenance of free-running rhythmicity (Helfrich-Förster, 1998; Renn et al., 1999). The ventral lateral neurons secrete the neuropeptide Pigment Dispersing Factor (PDF), which is necessary for rhythmicity under constant conditions (DD 25°C) (Renn et al., 1999). s-LNv secrete PDF in the dorsal part of the brain through their axonal terminals, henceforth referred to as dorsal projections (DP) (Park et al., 2000); and most circadian neurons in the network are responsive to PDF *via* expression of its receptor PDFR (Hyun et al., 2005; Mertens et al., 2005; Shafer et al., 2008). PDF is shown to rhythmically accumulate in the s-LNv dorsal terminals both under LD and DD (Park et al., 2000). It is probably also secreted rhythmically, as evidenced by the high amounts of rhythmic fasciculation of the s-LNv dorsal terminals observed, coinciding with the peak of accumulation (Fernández et al., 2008). PDF functions as a synchronizing factor in the circadian network, with complex effects on downstream neurons, such as maintaining rhythmicity in some clock cells, including the s-LNv themselves and adjusting the phase of the molecular clock in some other cells (Yoshii et al., 2009). Lack of PDF or PDFR causes a 60%–70% reduction in rhythmicity of locomotor activity, with an accompanying dampening of molecular clock oscillations (Peng et al., 2003), suggesting that chemical modes of communication play major roles in synchronizing the *Drosophila* circadian network. Similar desynchronization in the network connectivity is also observed upon genetic perturbation of the s-LNv membrane potential by constitutive expression of a sodium channel, NaChBac (Nitabach et al., 2006) or an inward rectifier potassium channel, Kir 2.1 (Nitabach et al., 2002; Depetris-Chauvin et al., 2011). Expression of NaChBac in the s-LNv results in complex periodicities in activity-rest rhythms, whereas expression of Kir 2.1 results in arrhythmicity of the resulting activity-rest behaviour. While the results of these experiments are not very surprising, an important point to note here is that molecular oscillations of known circadian clock proteins in the soma of pacemaker neurons were not found to be affected by the expression of NaChBac. The resulting behavioural output is affected possibly because of desynchrony in the network caused by alteration in the levels or release of the neuropeptide PDF (Nitabach et al., 2006). This again suggests that although the molecular clocks in individual oscillators are ticking reliably, communication among oscillators in the circadian neuronal network is crucial in regulating behaviour. Similar connectivity among individual oscillators can also be observed in the mammalian circadian clock, the Suprachiasmatic Nucleus (SCN). Although individual SCN neurons isolated in a culture dish display circadian, cell-autonomous molecular rhythms, and spontaneous firing rate of membrane potential, these outputs are desynchronized, out of phase, and lack precision and robustness (Hastings et al., 2018). In contrast, isolated SCN slices display rhythms that are synchronous, precise and robust for many days in culture, suggesting that connectivity among the oscillators plays a key role in generating coherent rhythms (Patton et al., 2016). The SCN is also highly interconnected *via* several slow-acting neuropeptides and fast-acting neurotransmitters. Vasoactive Intestinal Polypeptide (VIP) plays similar roles as PDF in the mammalian circadian clock network. Mutant mice lacking VIP or its receptor VPAC2 display desynchronized rhythms in activity, cellular oscillations and electrical firing, underscoring the importance of this neuropeptide in the circuit (Harmar et al., 2002; Hastings et al., 2014) In addition to VIP, GABA secreted by the SCN neurons also acts as a synchronizing factor in the network. The role of GABA in the network is however more complex as it acts synergistically with VIP on the phase relationships among neurons in different SCN sub-regions, which is highly dependent on the external environmental conditions. The role of GABA was found to be particularly important for switching SCN network states to synchronize to different external photoperiods (Rohr et al., 2019). Apart from VIP and GABA, other neuropeptides such as Arginine Vasopressin (AVP), Prokineticin *etc.* also play important roles both in the persistence of rhythms under free-running conditions as well as for the synchronization of behaviour to external, cyclic cues. Although, the role of neurons were almost exclusively explored in understanding circadian timekeeping by the SCN, recent studies show that astrocytes in the region of the SCN also play active roles in regulating the timing of behaviour (Brancaccio et al., 2017; Tso et al., 2017; Brancaccio et al., 2019). SCN astrocytes secrete glutamate which acts on the neurons and this astrocyte-neuron communication axis is important for the persistence of rhythmicity in behaviour (Brancaccio et al., 2017). Thus, there is an emerging idea that circadian timekeeping is brought about by a complex interplay between SCN neurons, astrocytes, and the many different communication factors that connect them together and enable them to function as a network.

## Communication in neural circuits: Overview of electrical synapses

**FIGURE 1:**
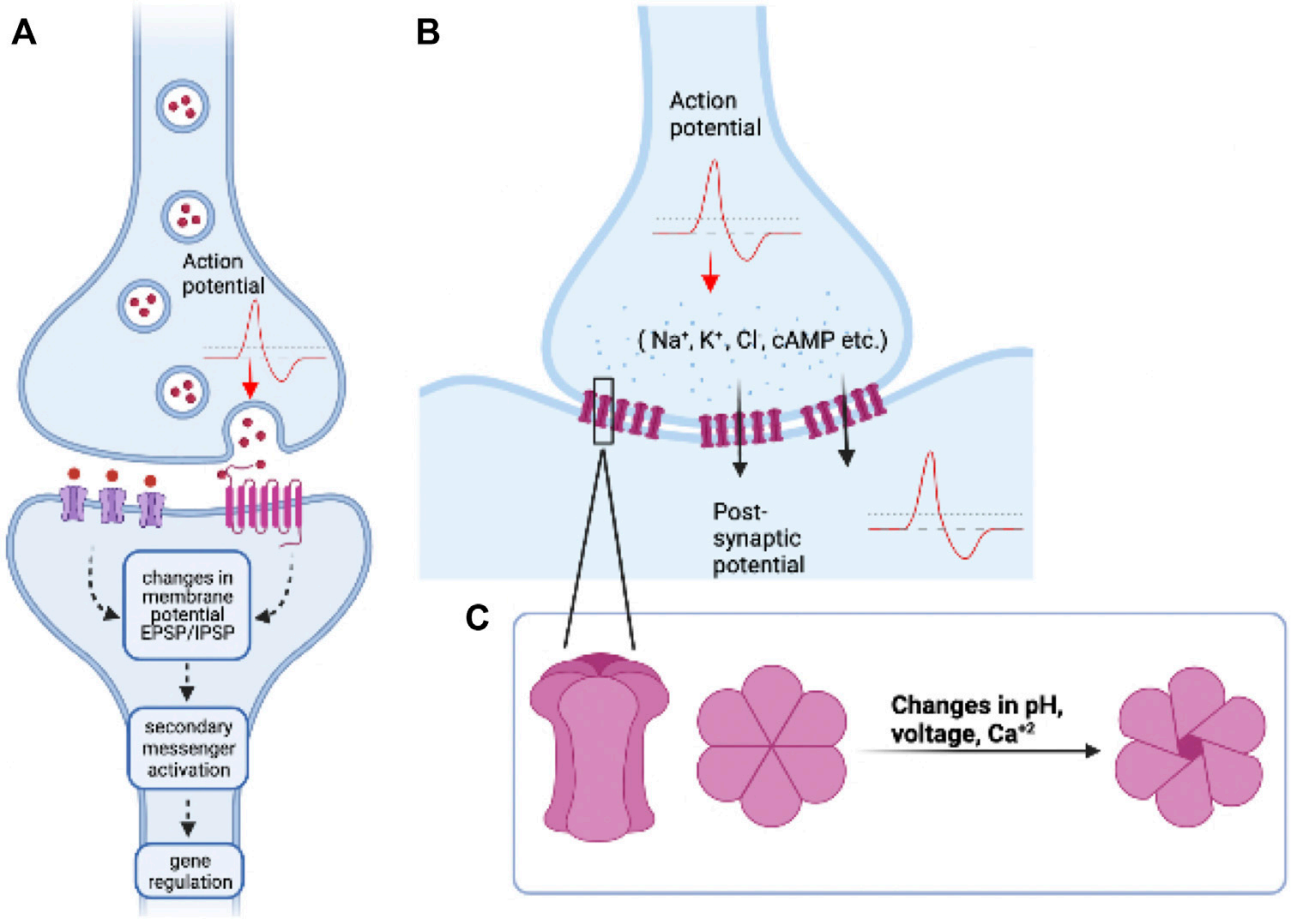
An overview of chemical and electrical synapses: **(A)** Chemical synapses work *via* the release of neurotransmitters or neuropeptides which bind to their respective ionotropic and/or metabotropic receptors and initiate a cascade of cell signalling events in the post-synaptic neuron. **(B)** Electrical synapses form direct cell-cell connections *via* gap junction proteins made up of Connexins or Innexins. The action potential/sub-threshold potential from the pre-synaptic cell, in this case, is directly transferred to the post-synaptic cell by passage of ions or small molecules, such that these cells are “electrically coupled.” **(C)** Inset: (left) Structure of a gap junction hemichannel (Connexon) made up of six units of Connexin or proteins. Top view of Connexon in closed (middle) and open (right) configurations. The opening and closing of gap junctions is regulated by various factors such as changes in pH, transmembrane voltage differences, and Ca^+2^ levels. Image created using BioRender.

Neural networks made up of neurons and glia are known to involve extensive inter-cellular communication. This is crucial for regulating several processes from development to behaviour and plasticity. Across organisms and behaviours, most studies have focused on the role of chemical synapses among neurons in a circuit, although electrical and chemical synapses have been known to co-exist in neural networks of most organisms (Pereda, 2014; Nagy et al., 2018). Electrical synapses also play major functional roles in regulation of several behaviours across organisms, and mutations in components of electrical synapses have been associated with several diseases and neurological disorders (Dere and Zlomuzica, 2012). While chemical synapses are made up of sophisticated molecular machinery where information is transferred at the synaptic clefts *via* the release of neuropeptides or neurotransmitters, electrical synapses are direct cell-cell connections made up of specialized structures called gap junctions (Figure 1). The first evidence of direct communication between cells *via* gap junctions was discovered in invertebrates. Furshpan and Potter recorded electrical activity from one-way synapses in the abdominal nerve cord of the crayfish (*Astacus fluviatilis*) and showed that action potentials pass directly between the giant axons to the motor neurons (Furshpan and Potter, 1957). Similar “electrical connections” were shown to be present in the lobster cardiac ganglion cells when electrodes were inserted into one cell, and recordings from the other cell showed an action potential of lowered amplitude and delayed time course (WATANABE, 1958). This form of electrical communication was thought to be mediated by direct connections between adjacent cells, called ‘nexus’ or ‘gaps.’ Electron microscopy also revealed the presence of these connections in several other tissues like smooth and cardiac muscles in mammals, rat epithelia, giant axon in the earthworm, mouse heart and liver cells *etc.* (Dewey and Barr, 1964; Revel and Karnovsky, 1967). However, there was no one-to-one correlation reported between the existence of these nexuses and electric coupling of these cells (Dewey and Barr, 1964), suggesting that these intercellular connections may have other roles to play in cell-cell coupling and adherence of cells. Although direct electrical connections between cells were first observed in invertebrates, the genes responsible for these cell-cell connections were first identified and isolated in vertebrates, and named as *Connexins*. The functionally analogous invertebrate *Innexin* genes were discovered much later, probably because of a lack of genetic sequence similarity between these two gene classes (Bauer et al., 2005). Gap junctions as we now know are clusters of intercellular channels made up of proteins called Connexins in vertebrates, Innexins in invertebrates, and Pannexins found in some chordates [reviewed in (Beyer and Berthoud, 2018)]. A cell may express one unit of the channel, known as the hemichannel, and two such hemichannels in adjacent cells can interact to form functional gap junctions. Gap junction hemichannels could be made up of the same class of Innexin proteins (homomeric) or different classes of Innexin proteins (heteromeric), similarly, gap junctions could be composed of the same class of hemichannels (homotypic) or different subunits of hemichannels (heterotypic) (Goodenough and Paul, 2009). Gap junctions, can be in the ‘open’ state or ‘closed’ state depending on the cellular needs and external and internal conditions including pH, voltage, calcium concentrations, and state of protein phosphorylation (Dbouk et al., 2009). They are usually present in cells as clusters on the cell membrane (called gap junctional plaques), and the recruitment and assembly of these proteins is a highly regulated process (Martin and Evans, 2004). There are over 20 *Connexin* genes in mammals and humans and several of the 25 *Innexin* genes are expressed in the fly *Drosophila melanogaster*, the leech *Hirudo medicinalis*, and the worm *Caenorhabditis elegans*. The various biochemical and physiological properties of gap junctions have been studied by expressing them in heterologous systems like paired *Xenopus* oocytes which do not express any of these proteins endogenously. These studies reveal that both Connexins and Innexins can only selectively form channels with certain other classes of Connexins or Innexins. The specific combinations determine the properties of these channels and are essential for their proper physiological functioning. However, it is worth noting that these specific combinations can change across developmental stages, across tissues, and with changes in external conditions and internal states, as seen with *C. elegans* electrical connectome, revealing the plasticity of electrical synapses (Bhattacharya et al., 2019).

Electrical synapses communicate with each other with little to no delay in the transmission rate (∼0.1 ms), which warrants its presence in networks controlling escape responses in some invertebrates (Edwards et al., 1999; Herberholz et al., 2002; Phelan et al., 2017). Electrical coupling is not just restricted to action potentials, but even to subthreshold currents like depolarization, hyperpolarization and, changes in membrane potentials (Faber and Pereda, 2018). Electrical synapses are not mere, passive conductors, but themselves contribute to electrical communication. Gap junctions have also been implicated in synchronous firing (either with a fast or slow time scales) of adjacent neurons in a network (Galarreta and Hestrin, 2001; Veruki and Hartveit, 2002; Curti et al., 2012). Although the functional significance of such firing synchrony is not very clear, some modelling studies (Lewis and Rinzel, 2000; Gansert et al., 2007) and experimental evidence have indicated that synchronous firing reduces noise in neuronal networks and facilitates the efficient release of hormones/neurotransmitters to drive downstream neurons (e.g.,- communication between rod cells and bipolar cells in the retina; (Attwell et al., 1987)) release of hormones in the locus coeruleus (Christie et al., 1989) and substantia nigra (Grace and Bunney, 1983). Such networks of synchronously firing neurons are also found in vertebrate motor systems (Li and Rekling, 2017).

Apart from the well-studied roles of gap junctions in the electrical coupling of cells, these proteins are also implicated in several non-channel based functions (Dbouk et al., 2009) like cell growth and migration (Kalra et al., 2006), cell division, and cell differentiation (Gu et al., 2003), cell signaling (Richard and Hoch, 2015) and, regulation of gene expression (Stains et al., 2003; Iacobas et al., 2004; Stains and Civitelli, 2005). Some gap junction proteins also function as hemichannels. These hemichannels are present in a ‘closed’ state on the cell membrane and their opening/closing are regulated by changes in membrane potential, ionic concentrations, metabolic state, and mechanical stimuli (Dbouk et al., 2009; Scemes et al., 2009). Hemichannels regulate the transport of small molecules like ATP and NAD + between the cell and the extracellular space. They are also involved in the regulation of calcium signalling, cell signalling, and differentiation (Belliveau et al., 2006; Evans et al., 2006), and apoptosis (Hur et al., 2003).

## Functional roles of electrical synapses in circadian neuronal networks

There are myriad functions played by electrical synapses in vertebrate and invertebrate neural circuits both during development of the circuit, and during the adult stages, however the detailed discussion of the mechanistic bases of these functions is beyond the scope of this article. There are several detailed and recent reviews summarizing the general functions of electrical synapses in neural circuits (O’brien, 2014; Haas et al., 2016; Connors, 2017; Alcamí and Pereda, 2019; Curti et al., 2022; Vaughn and Haas, 2022), but in the present review we will focus on the known roles for electrical synapses in circadian timekeeping circuits.

### Electrical synapses in the suprachiasmatic nucleus

**FIGURE 2:**
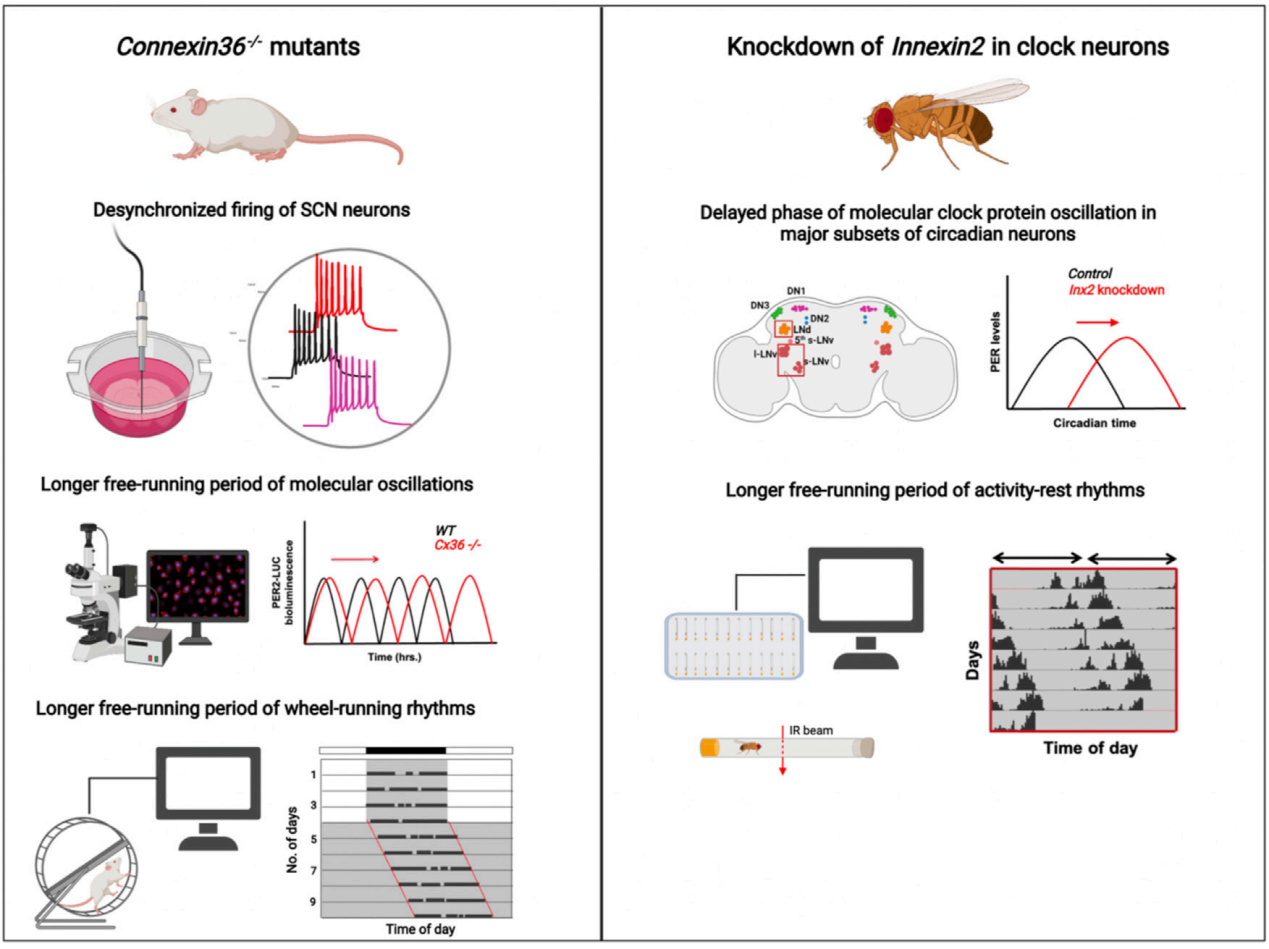
Summary of the circadian phenotypes observed in *Connexin36* mutant mice (left) and *Innexin2* mutant *Drosophila* (right): (left) Electrophysiological recordings from SCN slices of *Cx36* ^*−/−*^ mice show desynchronized firing of SCN neurons (Long et al., 2005)**,** PER2-Luc rhythms in *Cx36* ^*−/−*^ mice (red traces) oscillate with a longer free-running period compared to WT mice (black traces) over days (Diemer et al., 2017), and the wheel-running activity of adult mice with *Connexin36* mutation runs with a longer free-running period compared to WT mice under constant darkness (Long et al., 2005; Diemer et al., 2017) (right). Knockdown of *Innexin2* in clock neurons delays the phase of PER protein oscillation in major clock neuronal subsets in the *Drosophila* brain and lengthens the free-running period of activity-rest rhythms (Ramakrishnan and Sheeba, 2021). Image created using BioRender.

There is limited information about a role for gap junctions in circadian circuits and it is based on studies carried out in mammals. Perhaps the earliest evidence of non-synaptic coupling among neurons in the Suprachiasmatic nucleus (SCN), the central clock in mammals, came from studies on the development of fetal SCN. It was observed that rat fetal SCN show rhythms in glucose metabolism and neuronal firing as early as embryonic day E19 and E22, respectively (Reppert and Schwartz, 1984; Shibata and Moore, 1987), while synaptogenesis in the SCN happens much later postnatally between P4-P10 (Moore and Bernstein, 1989). This suggests that SCN behaves as a functional oscillator even before the synapses are completely developed in these animals, thus giving rise to the question of how these cells are coupled before chemical synapses are formed. An early study looked at neuronal firing rhythms in rat SCN neuronal cultures in a Ca^+2^-free medium which blocks synaptic transmission and found that these neurons fire synchronously even in the absence of synaptic transmission, suggesting that some form of coupling mechanism other than chemical transmission exists in the SCN (Bouskila and Dudek, 1993). Electrical synapses could be a potential mechanism by which these cells are coupled to each other and studies that followed started looking for evidence which suggests presence of gap junctions. While previous reports have shown that gap junctions abundantly couple SCN astrocytes, there were no reports of gap junctional coupling among the SCN neurons (Welsh and Reppert, 1996). However, this could be because the dissociation and culturing procedures could make the low density of gap junctional proteins present on the surface of neurons more scarce and undetectable in the background of astrocytes which are abundant in number. Hence, other studies examined if adult rat SCN tissues are coupled *via* gap junctions by injecting a tracer molecule, Neurobiotin, and tracing its passage through the cells in the tissue (Jiang et al., 1997). The authors reported that about 30% of SCN neurons show dye coupling. Furthermore, they show that these neurons show synchronous oscillations of membrane potential and voltage detected using electrophysiological recordings, suggesting that possibly the neurons in SCN are also coupled *via* gap junctions (Jiang et al., 1997). A later study complemented this by systematically examining the dye coupling among neurons in the rat SCN using a different tracer molecule, biocytin. Dye filling experiments with biocytin revealed that about 73% of SCN cells showed dye coupling, which was abolished on bath application of known gap junction blockers, strongly suggesting that these cells were indeed coupled by gap junctions (Colwell, 2000). Furthermore, these cells show time-of day dependent differences in dye coupling such that the cells are more coupled to each other during daytime than night time both under Light: Dark (LD) as well as constant darkness (DD), indicating that this preferential coupling is under the control of the circadian clock. Using antibodies against Connexins, they also show that SCN neurons show positive immunoreactivity to Connexin32 antibody, whereas Connexin43 was found abundantly present in SCN astrocytes (Colwell, 2000). Connexin32 and Connexin36 gap junctions were shown to be present in both rat and mice SCN slices using fluorescence and freeze-fracture electron microscopy (Rash et al., 2007). While immunocytochemical and physiological studies indicated the presence of gap junctions in the SCN, there were no reports on the functional roles played by gap junctions in circadian behaviour. The first evidence indicating the functional importance of gap junctions recorded behavioural rhythms along with electrophysiological recordings of the SCN neurons in *connexin36 (Cx36* ^*−/−*^ *)* mutant mice*,* (Long et al., 2005). *Cx36* ^−/−^ mice show defects in synchronous firing of neurons, with SCN from wild-type mice showing significantly higher synchrony in the firing of action potentials, suggesting that gap junctions composed of Cx36 are involved in the electrical coupling of SCN neurons (Long et al., 2005) (Figure 2). Further, they record the wheel-running behaviour of both wild-type and *Cx36* mutant mice under both entrained (LD) and free-running (DD) conditions. The rhythm properties were not observed to be significantly different among the genotypes under LD. Under DD, however, the mutant mice have significantly reduced circadian amplitude, low consolidation of activity (activity is dispersed over both day and night), and a transient but significantly lengthened period of free-running rhythms (Figure 2). (Long et al., 2005). Similarly, another study examined the requirement of gap junctions for synchrony in calcium oscillations among the SCN neurons (Wang et al., 2014b). They found that application of a known gap junction blocker, Carbenoxolone, in the bath when recording from slices, decreases the synchronous activity of neurons as measured by two photon imaging experiments, which also indicates that gap junctions are required for synchronous activity of SCN cells (Wang et al., 2014b). A more recent study re-examined the *Cx36* mutants used in Long et al., 2005 to understand the changes happening at molecular and cellular levels in these mice. This report shows that the desynchrony observed at the level of electrical coupling in *Cx36* mutants is not seen at the level of molecular oscillations measured by PER-luciferase imaging of SCN slices. The PER protein oscillations, albeit lengthened, appears to oscillate in-phase in all the cells in the SCN, and there is no desynchrony in the network (Figure 2). They observe that the behavioural period and the period of PER oscillations of *Cx36* mutants are slightly lengthened as compared to the wild-type mice, suggesting that absence of *Cx36* lengthens the behavioural period without affecting the synchrony of molecular oscillations of the cells in SCN (Diemer et al., 2017) (Figure 2). Apart from neuronal coupling, recently the role of Connexin43 (Cx43) for communication between astrocytes and neurons in the SCN was reported (Brancaccio et al., 2019). Cx43 is known to be highly expressed in astrocytic cells; blocking Cx43 hemichannels using an inhibitor interferes with the paracrine release of gliotransmitters including ATP and glutamate. This results in a reduced amplitude and delay in the period of *Per2-Luc* oscillations in the SCN network, emphasizing the importance of gap junctions in the glia-neuron communication axis in the SCN (Brancaccio et al., 2019). In summary, although there are many studies which report the presence of gap junctions in the SCN and describe its functional roles in the circuit and behavioural levels, our understanding of how gap junctions modulate circadian behaviour is very preliminary. There are very few, to no reports where each of the Connexin classes are systematically eliminated, and its effect on behaviour are assessed. Moreover, there are no studies that examine the mechanisms of how gap junctions may modulate circadian behaviour in mammals.

### Electrical synapses in the invertebrate clock network

Compared to vertebrates, there are even fewer studies which investigate the roles of electrical synapses in circadian circuits of invertebrates. An early study investigates the role of gap junctions in the circadian network of the cockroach, *Leucophaea maderae*. The use of gap junction blockers eliminates the synchronous firing of neurons in the accessory medulla region, which is the circadian pacemaker centre in insects (Schneider and Stengl, 2006). This observation was similar to the one in mammals where knockout of *connexin36* results in desynchronized firing among the SCN neurons (Long et al., 2005). Thus, it appears that gap junctions in circadian neurons of both vertebrates and invertebrates perform similar functions of synchronizing the electrical activity in these neurons, keeping in mind that perhaps more evidence is required to generalize the functions of these proteins across organisms and taxa. While the genetic, molecular and neuronal mechanisms of circadian rhythms have been well-studied in *Drosophila melanogaster* for about 3 decades, surprisingly, there is no systematic investigation of the roles played by electrical synapses in regulating rhythm properties. A study by Cao and Nitabach provides some evidence of the presence of gap junctions in the large ventral lateral neurons (l-LNv) in *Drosophila*. They show that recording the membrane potential from the l-LNv cells after application of a gap junction blocker Carbenoxolone in the bath, reduces the frequency of firing of action potentials in these cells (Cao and Nitabach, 2008). While this experiment suggests the presence of gap junctions among these neurons, it does not provide any information about the identity of the gap junction protein present in these cells or what the specific roles are (if any) in the regulation of circadian rhythm properties. A recent study from our group reports the role of specific gap junction proteins in the circadian circuit of *Drosophila melanogaster*. A genetic knockdown screen of all the eight *Innexin* genes in the clock neurons reveals the importance of Innexin1 and Innexin2 in modulating the free-running period (Ramakrishnan and Sheeba, 2021). We show that the knockdown of gap junction genes *Innexin2* and *Innexin1* lengthens the free-running period of activity-rest rhythms (Figure 2). Innexin2 protein was shown to be present in the small and large ventral lateral neurons (s-LNv and l-LNv respectively). Knockdown of *Innexin2* delays the molecular clocks in most circadian neurons (Figure 2) and increases the levels and amplitude of oscillation of the circadian neuropeptide, Pigment Dispersing Factor (PDF), which could ultimately explain the lengthening of free-running period seen in behavioural rhythms (Ramakrishnan and Sheeba, 2021). While this study provides a direct role for gap junction proteins in regulating a core clock property, much of the mechanism of how these proteins affect the molecular clock and the free-running period remains unknown. Do *Innexin1* or *Innexin2* mutant flies exhibit time-of-day dependent oscillations in membrane potential in the LNv? Does knockdown of *Innexin1* and *Innexin2* affect the absolute membrane potential value of LNv as compared with controls or does it affect the synchronous firing of those neurons? These are some of the questions that need to be addressed to better understand the mechanisms by which gap junctions affect circadian rhythms.

## Methods to visualize electrical synapses

**FIGURE 3:**
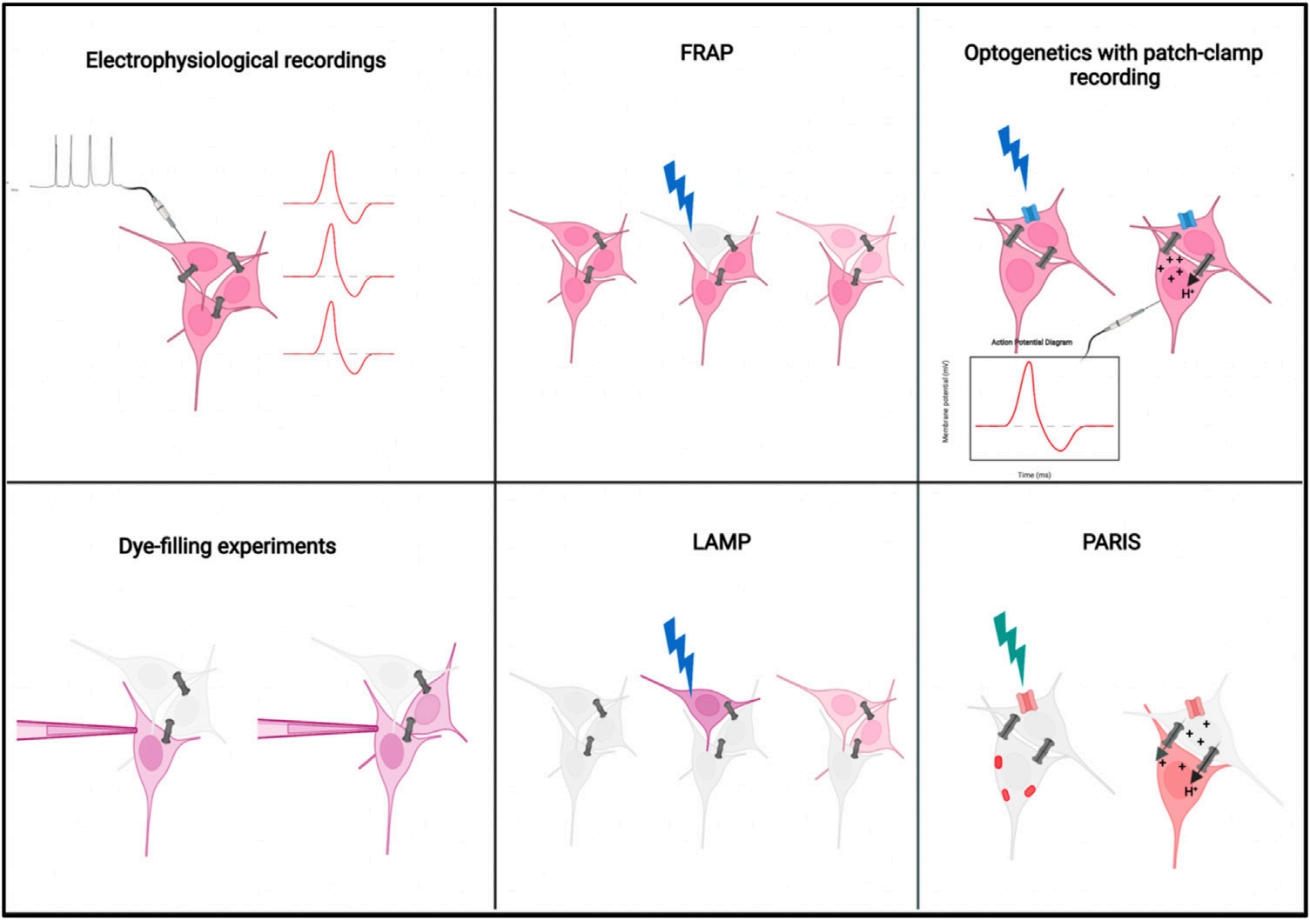
Methods to visualize electrical synapses: The schematic represents the methods currently available to visualize electrical synapses in the nervous system. Electrophysiological and dye-filling methods are invasive and technically challenging. FRAP and LAMP measure the rate of transfer of fluorescent molecules, hence can be quantitative, but could cause tissue damage. The recent improved methods utilize genetically encoded indicators including channel rhodopsins for optogenetics and ArchT and pHluorin for PARIS. Improvements that address shortcomings for these techniques should help improve the detection of functional electrical synapses among cells. Detailed descriptions of each technique are provided in the text. FRAP, Fluorescence Recovery After Photobleaching; LAMP, Local Activation of Molecular Probe; PARIS, Pairing Actuators and Receivers for optically ISolating gap junctions. Image created using BioRender.

As compared to methods for assaying functional connectivity across chemical synapses, there are fewer reliable methods available to visualize the functional electrical synapses between cells. Early studies which report the presence of gap junctions between cells relied on electron microscopy techniques. Although electron micrographs can provide information about the anatomical presence of gap junction between cell types, it does not indicate functional connectivity among them. Moreover, it is tedious to perform electron microscopy, requires advanced technical expertise, and is unsuitable for most tissue types. A definite way of measuring electrical connectivity among cell types is to perform paired electrophysiological recordings (Figure 3). However, it is technically challenging, invasive, and not suitable for all types of cells and tissues as it may not be possible to conduct whole-cell recordings from neurons or processes located in certain regions of the brain or other parts of the nervous system. A similarly invasive method which is commonly used to confirm electrical connectivity among cells are dye microinjections (Figure 3). The logic behind these experiments is to examine if cells are coupled *via* gap junctions by injecting a tracer molecule, like neurobiotin or biocytin, and tracing its passage through the cells in the tissue. (Jiang et al., 1997). Since tracers are small molecules (molecular weight <1,000 Da) that can travel from one cell to another *via* gap junctions, if one cell is injected with the tracer and is coupled to the other cell *via* gap junctions, then over time one could observe Neurobiotin in the coupled cell as well. Although this is a clear technique to assess functional gap junction coupling, it has problems similar to electrophysiological recordings, i.e. inaccessibility of certain cell types to dye filling, invasive and technically challenging. Dye diffusion is an irreversible process and so the same gap junctions cannot be repeatedly measured to evaluate their dynamics, regulations and plasticity. Other ways of visualization of electrical synapses which are relatively non-invasive include Fluorescence Recovery After Photobleaching (FRAP) (Wade et al., 1986) and Local Activation of Molecular fluorescent Probes (LAMP) (Dakin et al., 2005) (Figure 3). FRAP relies on incubating the cells with a small, cell-permeable fluorescent molecule like Fluorescein. The cells are then photobleached with a powerful laser beam and the rate of recovery of fluorescence in the bleached cell due to transfer of fluorescein molecules from neighbouring, unbleached cells *via* gap junctions is calculated. The advantages of this technique are its relative non-invasiveness compared to electrophysiology and dye-filling and the ability to calculate “strength of gap junctional coupling” between cells by measuring the rate of recovery after photobleaching. The disadvantages are the tissue damage caused by the use of a high intensity laser beam and the lack of cell-type specificity which limits its use to homogenous cell cultures. LAMP is similar to FRAP, but overcomes the disadvantage of phototoxicity caused by FRAP, by using directed UV light to uncage a molecule NPE-HCCC2-AM which emits blue fluorescent light upon uncaging. The rate of transfer of this fluorescent molecule *via* gap junctions to neighbouring cells is then measured and quantified. The disadvantage of this technique includes the lack of cell-type specificity limiting its application *in vivo*. Moreover, the uncaging of the molecule is irreversible thus making it difficult to study the dynamics of gap junctions over prolonged periods. A significant technical advance to visualize electrical synapses between cells came about with the use of genetically encoded fluorescent sensors. Initial attempts in this direction was made using a novel genetically encoded fluorescent sensor called Pado which is composed of a voltage sensor and a pH sensitive fluorescent indicator which detects the transfer of protons between adjacent cells *via* gap junctions (Kang and Baker, 2016). Optogenetics was also modified and utilized along with electrophysiology for detection of electrical synapses across neurons in the *Drosophila* olfactory system (Wang et al., 2014a) (Figure 3). The idea was to express a Channelrhodopsin in one cell, activate it *via* light and perform patch-clamp recordings to record changes in membrane potential from another cell which could be coupled to the first one *via* gap junctions. To distinguish the signal from the chemical synapse mediated communication, one may have to use appropriate gap junction mutants as controls as well as record after application of antagonists of the neurotransmitters or neuropeptides used by the cell of interest. However, the involvement of electrophysiology could make the technique tedious and technically challenging and hence limit its usage in many cells and tissues. The most recent addition to this list comes in the form of PARIS (Pairing Actuators and Receivers to optically ISolate gap junctions) (Figure 3). PARIS uses a ligand gated proton pump ArchT (referred to as actuator) in combination with a pH indicator like pHluorin (referred to as receiver). The actuator and receiver are expressed by adjacent cells which are being assayed for the presence of gap junctions. Activation of the actuator by light allows the transfer of protons between these adjacent cells, which changes the pH of the neighbouring cell which is detected by the receiver and indicated by a change in fluorescence. This has been shown to work in multiple cell types in culture as well as in *Drosophila* central nervous system (Wu et al., 2019). It is a technique that overcomes major limitations of the previous ones. All the components are genetically encoded, hence it is non-invasive to the cell or tissue, it is reversible and cell-type specific. One major shortcoming of this technique is the inability to assess gap junctional coupling among homogenous cell types which are targeted by the same genetic drivers. Additionally, it requires a prior assumption of potentially coupled cells that one must target with specific drivers. At this time there is no method that can detect coupling *via* gap junctions at neurites to the soma. Improvements to addresses these shortcomings would increase the adaptability of this technique across multiple model systems.

## Discussion and concluding remarks

In this review, we summarize the importance of electrical synapses in neuronal circuits with a special emphasis on the well-characterised circadian circuits of mammals and invertebrates. While various aspects of the neuronal circuitry governing circadian behaviour are well-studied since the past few decades, a crucial component of connectivity mediated by electrical synapses in these circuits are being left out. Although there are independent reports in some invertebrate and vertebrate model systems on the roles played by electrical synapses in regulating circadian behaviour, molecular oscillations, or both, there is a lack of systematic investigation into the underlying mechanisms. A major limiting factor for this could be the technical difficulties in visualization and reliable interpretation of the presence and function of electrical synapses among cells. Over the past decade or so, intersection of newer genetic and visualization techniques have led to the development of efficient tools to analyse the presence of electrical synapses among cells. With the development of these tools, one hopes that there is better appreciation for the roles of electrical synapses in the functioning of neuronal circuits, not just limited to circadian circuits, but in other behaviours as well.

One could make several hypotheses regarding the mechanisms by which electrical synapses function in circadian networks. Mutants lacking components of Connexins or Innexins have very similar behavioural phenotypes in mammals and invertebrates respectively, i.e., desynchronized firing of neurons, delay in the phase/longer period of molecular clock oscillations and lengthening of free-running period of behavioural rhythms. This could suggest that electrical synapses play similar roles in regulating circadian rhythms across organisms. Since gap junctions are required for synchronous firing of clock neurons, an immediate question that comes to mind is the relevance of such synchronized firing. Although there are no clear answers to this question with respect to circadian circuits, studies from other systems have shown that a set of pre-synaptic neurons are most efficient in sending information to post-synaptic sites when their firing is synchronized. Synchronized firing could lead to reduction in noise in neural circuits and efficient release of neurotransmitters/neuropeptides (Connors, 2017). Most of the SCN neurons release neuropeptides like VIP, AVP, GRP, or neurotransmitters like GABA. Similarly, Innexin2 was found to be present in the s-LNv which release the neuropeptide PDF in the circadian network. Thus, it would be pertinent to ask if the efficiency of release of these neuropeptides and transmitters is affected in animals lacking Connexin or Innexin, which could ultimately be reflected in the behavioural differences seen in case of mutants.

An alternate hypothesis would be the involvement of electrical synapses in regulating membrane properties of clock neurons. Gap junctions allow passage of ions and small molecules from cell to cell or cell to extracellular milieu, thus coupling them together. A disruption of these processes could affect the membrane properties of these neurons, thus affecting the molecular clock and behaviour. Membrane potentials vary in a time-of-day dependent manner in both invertebrates and mammals, such that the resting membrane potential is more depolarized during the day *versus* night (Sheeba et al., 2008) (Cao and Nitabach, 2008; Colwell, 2011). Genetic manipulations that affect membrane firing rates and potentials such as constitutive depolarization and hyperpolarization, as well as those that affect the daily rhythms in membrane firing, directly affect the molecular clocks, suggesting a tight link between these two processes (Nitabach et al., 2002; Nitabach et al., 2006; Colwell, 2011; Mizrak et al., 2012). Although mutation in gap junction genes affect the phase and/or period of molecular clock oscillations in both vertebrates and invertebrates, the mechanistic basis of these changes are unclear. Further investigation is required to link the roles of electrical synapses to changes in membrane potentials, which could in turn affect the molecular clocks. Many gap junction genes also function as hemichannels and could be involved in the transfer of small molecules and secondary messengers like cAMP. Mutations in gap junction genes could also affect these processes, which in turn can disrupt the cellular signalling pathways and the molecular clock oscillations.

Apart from coupling neuronal cells with each other, gap junctions could also be involved in communication among glial cells themselves or between glial cell and neurons. A study in mammals demonstrated the importance of Connexin43 in communication between astrocytes and neurons in the SCN. Connexin43 functions as hemichannels in astrocyte, which is required for efficient release of the gliotransmitter glutamate, and a lack of these channels affects the period of molecular clock oscillations and behaviour in mice (Brancaccio et al., 2019). Thus gap junctions could potentially function as a communication axis between astrocytes and neurons to regulate clock functions and sleep.

Finally, a recent study in *C. elegans* maps the expression pattern of all the Innexins expressed in each of its neurons (Innectome) under normal and dauer (induced by unfavourable environments like starvation) states, and found that the Innectome profile responds to changing external environments (Bhattacharya et al., 2019). This suggests that electrical synapses can exhibit remarkable amount of plasticity, similar to chemical synapses and this is probably important for organisms to adapt to changing external environments. With developments in methods such as single-cell RNA sequencing, it would not be difficult to obtain the expression profiles of Connexins or Innexins in the clock neurons, and it would be interesting to determine if these profiles change with changing external conditions. A report which dissects the chemical connectome of the clock neuronal network in *Drosophila* finds remarkable insights into coupling mechanisms among clock neurons (Shafer et al., 2022). A similar analysis of coupling mediated by electrical synapses in the clock network would go a long way in understanding the roles of these proteins in regulation of circadian rhythms and sleep.
